# Consensus, uncertainties and challenges for perennial bioenergy crops and land use

**DOI:** 10.1111/gcbb.12488

**Published:** 2017-11-27

**Authors:** Jeanette Whitaker, John L. Field, Carl J. Bernacchi, Carlos E. P. Cerri, Reinhart Ceulemans, Christian A. Davies, Evan H. DeLucia, Iain S. Donnison, Jon P. McCalmont, Keith Paustian, Rebecca L. Rowe, Pete Smith, Patricia Thornley, Niall P. McNamara

**Affiliations:** ^1^ Centre for Ecology & Hydrology Lancaster Environment Centre Lancaster LA1 4AP UK; ^2^ Natural Resource Ecology Laboratory Colorado State University Fort Collins CO 80523‐1499 USA; ^3^ Global Change and Photosynthesis Research Unit USDA‐ARS and Department of Plant Biology University of Illinois Urbana IL 61801 USA; ^4^ “Luiz de Queiroz” College of Agriculture University of São Paulo Avenida Pádua Dias 11‐13418‐900 Piracicaba Brazil; ^5^ Department of Biology, Research Centre of Excellence on Plants and Ecosystems University of Antwerp B‐2610 Wilrijk Belgium; ^6^ Shell International Exploration and Production Inc. Shell Technology Centre Houston Houston TX 77082 USA; ^7^ Institute of Biological, Environmental and Rural Sciences (IBERS) Aberystwyth University Aberystwyth SY23 3EQ UK; ^8^ Department of Soil and Crop Sciences Colorado State University Fort Collins CO 80523‐1499 USA; ^9^ Institute of Biological & Environmental Sciences University of Aberdeen Aberdeen AB21 3UU UK; ^10^ Tyndall Centre for Climate Change Research School of Mechanical, Aerospace and Civil Engineering University of Manchester Manchester M13 9PL UK

**Keywords:** biofuels, biomass, greenhouse gas emissions, land‐use change, life‐cycle assessment, nitrous oxide, perennial bioenergy crops, soil carbon

## Abstract

Perennial bioenergy crops have significant potential to reduce greenhouse gas (GHG) emissions and contribute to climate change mitigation by substituting for fossil fuels; yet delivering significant GHG savings will require substantial land‐use change, globally. Over the last decade, research has delivered improved understanding of the environmental benefits and risks of this transition to perennial bioenergy crops, addressing concerns that the impacts of land conversion to perennial bioenergy crops could result in increased rather than decreased GHG emissions. For policymakers to assess the most cost‐effective and sustainable options for deployment and climate change mitigation, synthesis of these studies is needed to support evidence‐based decision making. In 2015, a workshop was convened with researchers, policymakers and industry/business representatives from the UK, EU and internationally. Outcomes from global research on bioenergy land‐use change were compared to identify areas of consensus, key uncertainties, and research priorities. Here, we discuss the strength of evidence for and against six consensus statements summarising the effects of land‐use change to perennial bioenergy crops on the cycling of carbon, nitrogen and water, in the context of the whole life‐cycle of bioenergy production. Our analysis suggests that the direct impacts of dedicated perennial bioenergy crops on soil carbon and nitrous oxide are increasingly well understood and are often consistent with significant life cycle GHG mitigation from bioenergy relative to conventional energy sources. We conclude that the GHG balance of perennial bioenergy crop cultivation will often be favourable, with maximum GHG savings achieved where crops are grown on soils with low carbon stocks and conservative nutrient application, accruing additional environmental benefits such as improved water quality. The analysis reported here demonstrates there is a mature and increasingly comprehensive evidence base on the environmental benefits and risks of bioenergy cultivation which can support the development of a sustainable bioenergy industry.

## Introduction

The global use of biomass for energy production has increased rapidly in response to the introduction of renewable energy mandates, particularly in the United States and the European Union (110th Congress of the United States 2007, Council Directive 2009/28/EC). These mandates were introduced to support domestic energy security and mitigate the climate change impacts of transportation by reducing reliance on fossil fuels. More broadly, ‘bioenergy’ refers to the delivery of heat, electricity or transport fuels from a diverse portfolio of biomass feedstocks processed through a range of conversion technologies, with significant potential for greenhouse gas (GHG) emission reductions compared to fossil fuels (Creutzig *et al*., 2015). Many climate stabilization scenarios suggest that the wide‐scale deployment of bioenergy systems augmented with carbon capture and storage (BECCS) will be necessary to correct emissions overshoot and keep future atmospheric GHG concentrations at levels below that implied in the <2 °C target (430–480 ppm CO_2_‐eq) (Kriegler *et al*., 2015; Riahi *et al*., 2015; Smith *et al*., 2016). However, there are sustainability concerns related to the expansion of bioenergy feedstock cultivation globally, such as potential conflicts with food production through direct (dLUC) and indirect land‐use change (iLUC), excessive nitrous oxide (N_2_O) emissions due to fertilizer application and land disturbance, and impacts on land and water resources, including soil organic carbon stocks (hereafter referred to as soil carbon), which could result in undesired outcomes (Crutzen *et al*., 2008; Smith & Searchinger, 2012; DeCicco, 2013).

The environmental costs and benefits of bioenergy have been the subject of significant debate, particularly for first‐generation biofuels produced from food (e.g. grain and oil seed). Studies have reported life‐cycle GHG savings ranging from an 86% reduction to a 93% increase in GHG emissions compared with fossil fuels (Searchinger *et al*., 2008; Davis *et al*., 2009; Liska *et al*., 2009; Whitaker *et al*., 2010). In addition, concerns have been raised that N_2_O emissions from biofuel feedstock cultivation could have been underestimated (Crutzen *et al*., 2008; Smith & Searchinger, 2012) and that expansion of feedstock cultivation on agricultural land might displace food production onto land with high carbon stocks or high conservation value (i.e. iLUC) creating a carbon debt which could take decades to repay (Fargione *et al*., 2008). Other studies have shown that direct nitrogen‐related emissions from annual crop feedstocks can be mitigated through optimized management practices (Davis *et al*., 2013) or that payback times are less significant than proposed (Mello *et al*., 2014). However, there are still significant concerns over the impacts of iLUC, despite policy developments aimed at reducing the risk of iLUC occurring (Ahlgren & Di Lucia, 2014; Del Grosso *et al*., 2014).

In contrast to annual crops, bioenergy from dedicated perennial crops is widely perceived to have lower life‐cycle GHG emissions and other environmental cobenefits (Rowe *et al*., 2009; Creutzig *et al*., 2015). Perennial crops such as *Miscanthus* and short‐rotation coppice (SRC) willow and poplar have low nitrogen input requirements (with benefits for N_2_O emissions and water quality), can sequester soil carbon due to reduced tillage and increased belowground biomass allocation, and can be economically viable on marginal and degraded land, thus minimizing competition with other agricultural activities and avoiding iLUC effects (Hudiburg *et al*., 2015; Carvalho *et al*., 2017). With respect to the perennial crop sugarcane, large GHG savings can be achieved due to high crop productivity and the use of residues for cogeneration of electricity, whilst the recent shift to mechanized harvest without burning in Brazil should also increase the potential for soil carbon sequestration (Silva‐Olaya *et al*., 2017). Nevertheless, the site‐level impacts of perennial crop cultivation on ecosystem carbon storage (resulting from dLUC) vary geographically, dependent on soil type and climate (Field *et al*., 2016). In addition, land management decisions and the type of land converted to bioenergy crop production have variable effects on soil carbon stocks and N_2_O emissions which are difficult to quantify accurately (Gauder *et al*., 2012; Palmer *et al*., 2014; Qin *et al*., 2016), leading to large uncertainties in the life‐cycle GHG balance of bioenergy (Rowe *et al*., 2011; Njakou Djomo & Ceulemans, 2012; Davis *et al*., 2013). These uncertainties create a complex picture for policymakers to assess the most cost‐effective and environmentally sustainable options for bioenergy deployment.

Over the last decade, a considerable body of field, laboratory and modelling research has addressed uncertainties in the dLUC and N_2_O implications of perennial bioenergy crop cultivation but has often reported contradictory evidence. To address this lack of clarity, a workshop was convened in 2015 with leading researchers, policymakers and industry/business representatives from the UK, EU, and internationally as part of the Ecosystem Land‐Use Modelling and Soil Carbon GHG Flux Trial (ELUM; Harris *et al*., 2014). The workshop aimed to: compare outcomes from global research on the cycling of carbon, nitrogen and water in perennial feedstock‐producing systems; identify consensus in conclusions drawn; highlight key uncertainties and knowledge gaps; and identify priorities for future research. The effects were considered across a range of scales (field, landscape and global) within the context of the whole life cycle of bioenergy production, with a focus on perennial cellulosic crops and sugarcane grown in the EU and North and South America, systems perceived to have the greatest potential to deliver significant GHG savings from bioenergy. Here, we discuss six consensus statements that summarize the current understanding of the environmental costs, benefits and trade‐offs of cultivating perennial bioenergy crops. These statements were formulated during the workshop through a process of facilitated discussion and reflection. To identify key areas of certainty, uncertainty and knowledge gaps, facilitated expert discussion was used to explore stakeholder perspectives and collate elicited ideas and questions into coherent themes. Certainties, uncertainties and knowledge gaps were then ranked and prioritized using an impact‐resolution difficulty matrix, placing issues on two axes of the potential benefit (low to high) vs. the difficulty to test/resolve (low to high). A common consensus was then established among stakeholders (research, policy and industry) on which consensus statements could be made. The strength of evidence for and against these statements was explored through consideration of exemplar projects during the workshop (see Acknowledgements), and through additional literature review and data analysis. The statements explored are as follows:

1. N_2_O emissions from perennial crops strongly depend on the previous land use with the greatest risk of large emissions during crop establishment.
2. Planting perennial bioenergy crops on low carbon soil will minimize soil carbon losses in the short‐term and promote soil carbon sequestration in the long‐term.
3. Variability in soil carbon stock changes influences the life‐cycle GHG balance of bioenergy production much more than variability in nitrogen‐related emissions over most common assessment timescales.
4. Perennial bioenergy crops can provide substantial climate mitigation when used to replace fossil fuels but land‐use tensions must be mitigated.
5. Perennial bioenergy crops marginally reduce water availability at landscape scale but improve water quality through reduced nitrate leaching.
6. Ecosystem process‐based models are essential for assessing bioenergy viability and environmental performance at landscape and regional scales, but they have only recently been applied to evaluate specific land‐use policies and strategies.

## Statement 1: N_2_O emissions from perennial crops strongly depend on the previous land use with the greatest risk of large emissions during crop establishment

**Figure 1:**
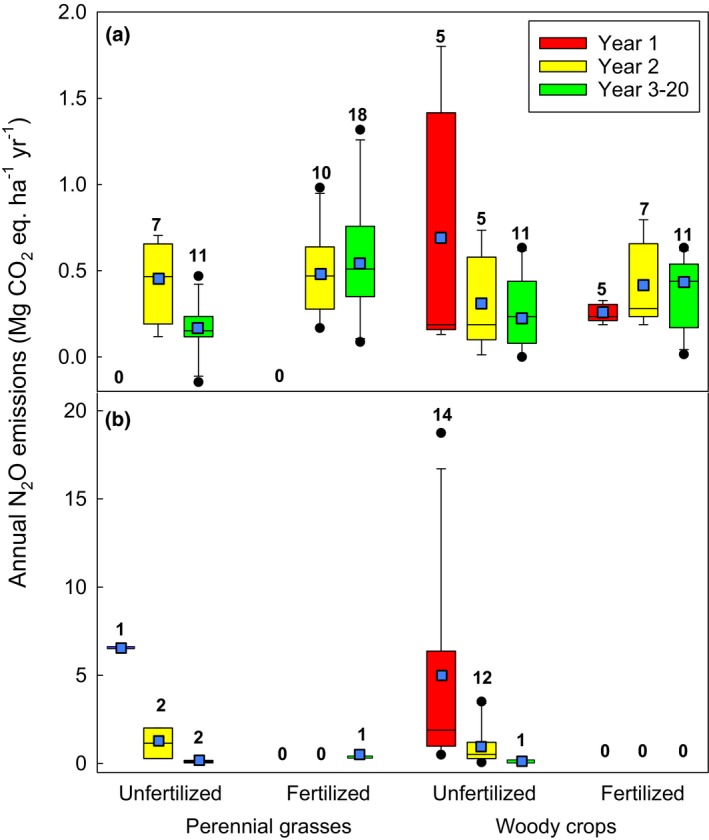
Effects of prior land‐use (a) annual crops and (b) grassland on annual N_2_O emissions of perennial grasses (*Miscanthus*, switchgrass) and woody crops (SRC willow and SRC poplar) grown with and without fertilizer. Box plot: the bottom and top of the box are the first and third quartiles, and the line within the box is the second quartile (median), 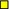 = average, whiskers indicate the 10th and 90th percentiles, dots indicate outliers. The values show the number of data sets. Note the *y*‐axis scales of (a) and (b) differ by an order of magnitude. Summary data are presented in Table S1.

When analysing the GHG balance of bioenergy production, fluxes of N_2_O from the soil need to be quantified due to their significant global warming potential (IPCC 2014). Until recently, it was assumed that N_2_O emissions made a minor contribution to the GHG balance of perennial bioenergy crops, due to the low or negligible amounts of fertilizer typically applied. However, empirical data were lacking (Jorgensen *et al*., 1997), creating a major uncertainty in calculating the GHG balance of bioenergy production (Rowe *et al*., 2011). We reviewed 28 publications from 2008 to 2016 (comprising 87 scenarios of crop/prior land‐use/fertilizer rate, Table S1) and showed that the magnitude of soil N_2_O emissions from perennial grasses (*Miscanthus*, switchgrass) and woody crops (SRC poplar and willow) varied significantly, dependent on historic and current fertilizer rates, prior land use (annual crops, grassland) and time since planting [establishment (yr. 1–2) and postestablishment (yr. 3+), Fig. 1].

In annual cropland converted to unfertilized perennial bioenergy crops, we found that annual average N_2_O emissions were 61% and 48% smaller in established crops (yr. 3+) compared with the crop establishment phase (yr. 1–2) for perennial grasses and woody crops, respectively (Fig. 1). In contrast, no postestablishment decline in N_2_O emissions was observed for fertilized perennial crops, likely due to repeated fertilizer applications (Fig. 1). For perennial crops planted onto grassland, very large annual N_2_O emissions were reported in the establishment phase of unfertilized perennial grasses and woody crops (Fig. 1; Nikiema *et al*., 2012; Palmer *et al*., 2014; J.P. McCalmont, unpublished data), but these declined by more than 95% in mature crops (Fig. 1; Table S2). Establishment phase emissions were intermittent and highly variable when aggregated on an annual basis, with emissions from SRC willow and poplar planted onto grassland ranging from 0.50 to 18.7 Mg CO_2_‐eq ha^−1^ yr^−1^ (Nikiema *et al*., 2012; Palmer *et al*., 2014).

Elevated N_2_O emissions during crop establishment in both grasslands and annual crops are likely caused by denitrification associated with high soil nitrate levels following soil tillage, herbicide application to remove existing vegetation, increased residue decomposition and/or fertilization of the previous crop (Palmer *et al*., 2014; Zenone *et al*., 2016). The significant differences in the magnitude of establishment phase emissions in crops planted onto grassland (Fig. 1) have been attributed to differences in soil nitrogen stock and wetness (Palmer *et al*., 2014). However, the small number of publications (six) on grassland conversion to bioenergy crops highlights a major knowledge gap, particularly for perennial grasses (Table S1).

Despite this variability in N_2_O emissions with prior land use, crop maturity, and fertilization rate, postestablishment emissions from perennial crops were generally much lower than emissions from annual crops. This was demonstrated in a small number of studies where average annual N_2_O emissions were twofold to 165‐fold greater in annual compared to adjacent perennial crops (Don *et al*., 2012; Drewer *et al*., 2012; Gauder *et al*., 2012; Gelfand *et al*., 2016). With respect to grasslands, no direct comparisons have been published. The intensity of grassland management and related N_2_O emissions vary widely, dependent on the rate and type of nitrogen inputs and the prevailing climate (temperature/moisture) (Cowan *et al*., 2015; Kelliher *et al*., 2017). For perennial crops planted onto intensively managed grassland, where nitrogen fertilizer and urine excretion by livestock result in high N_2_O emissions, we expect postestablishment emissions to be significantly lower, but empirical data are needed to quantify this.

N_2_O emissions in agricultural soils are highly variable in space and time. For example, in SRC poplar, 44% of total N_2_O emissions (over four years) occurred during one single peak following crop establishment (Zona *et al*., 2013b; Zenone *et al*., 2016), whilst 1.1% of the area of a Scottish grassland was responsible for 55% of the estimated daily N_2_O flux, measured during an intensive 72‐h sampling campaign (Cowan *et al*., 2015). Accurately quantifying and scaling emissions remain difficult due to the limitations of current measurement methodologies (Chadwick *et al*., 2014; Merbold *et al*., 2014). In 25 of the 28 bioenergy publications reviewed here, static chambers with noncontinuous (weekly or monthly) measurements were used which resulted in interpolated datasets with large temporal and spatial uncertainty (Table S1). Automatic chambers with high temporal, but low spatial resolution (Díaz‐Pinés *et al*., 2017) and eddy covariance (high temporal resolution which integrates spatial variability over a wide area) have also been deployed in a small number of studies. Whilst these methods also have limitations, current methodologies are useful in helping to better understand the sources of variability in the GHG balance of conventional and dedicated bioenergy crops and the influence of direct land‐use change and land management (Zona *et al*., 2013a; Cowan *et al*., 2015; Zenone *et al*., 2016).

## Statement 2: Planting perennial bioenergy crops on low carbon soils will minimize soil carbon losses in the short‐term and promote soil carbon sequestration in the long‐term

Increased rates of soil carbon sequestration in perennial bioenergy plantations have been widely proposed as a cobenefit of bioenergy production, contributing to the GHG mitigation potential of bioenergy (Hillier *et al*., 2009). Most perennial bioenergy feedstocks, particularly grasses, allocate a higher proportion of dry matter belowground, relative to annual crops. This higher carbon input tends to favour an increase in soil carbon stocks (Frank *et al*., 2004; Carvalho *et al*., 2017). However, large variation in the rates of soil carbon stock change (∆C) has been reported for land converted to perennial bioenergy crops, ranging from significant soil carbon sequestration to significant loss (Qin *et al*., 2016; Rowe *et al*., 2016; Richards *et al*., 2017). A range of interacting factors – including climate, soil texture, previous and/or current crop management intensity and changes in inputs – determines the effects of land‐use change on soil carbon stocks (Garten *et al*., 2011; McClean *et al*., 2015) making predictions of ∆C challenging. In addition, some early studies may have inflated the potential soil carbon sequestration benefit of perennial bioenergy due to the use of fixed depth sampling instead of bulk density‐corrected methodologies (Mello *et al*., 2014; Walter *et al*., 2015; Rowe *et al*., 2016).

**Figure 2:**
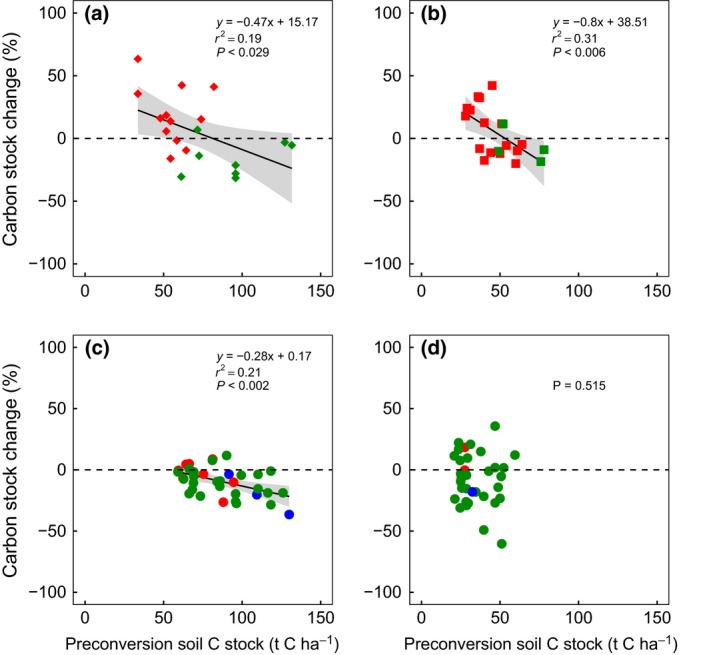
Relationship between preconversion soil carbon stock (^pre^C) and carbon stock change (∆C) following land conversion to (a) SRC willow UK; (b) SRC willow EU; (c) sugarcane with soil clay content <60%; and (d) sugarcane with soil clay content >60%. Colour indicates prior land use: red = annual crops, green = grassland and blue = natural vegetation (Cerrado). Data sources are as follows (a) Rowe *et al*. (2016), (b) Walter *et al*. (2015) (c) and (d) Mello *et al*. (2014). Plots show data with high leverage points removed.

Prior land use has been widely proposed as a key predictor of ∆C, with transitions from grassland to perennial bioenergy crops purported to have more detrimental effects on soil carbon stocks than transitions from annual crops (Don *et al*., 2012; Walter *et al*., 2015; Qin *et al*., 2016; Rowe *et al*., 2016). Yet within these broad land‐use classifications (annual crops, grassland), there is considerable variation in the magnitude and direction of ∆C reported following conversion to perennial bioenergy crops (Anderson‐Teixeira *et al*., 2009; Don *et al*., 2012; Walter *et al*., 2015; Qin *et al*., 2016; Rowe *et al*., 2016). Recent evidence has indicated that preconversion soil carbon stock (^pre^C) may be a better predictor of ∆C at regional scales than prior land use with a lower ^pre^C providing a greater opportunity for carbon accumulation (Rowe *et al*., 2016). Given the long timescales required to detect a change in ∆C, most studies employ a paired site approach where ^pre^C is derived from an adjacent piece of land representing the preconversion land use. Care is required in selecting such sites to represent the pre‐LUC situation as any variation in pairings will confound results. Using this approach, a significant negative correlation between ∆C and ^pre^C (0–30 cm depth) was identified for land converted to woody bioenergy crop cultivation, comparing 21 converted and unconverted sites in the UK (Fig. 2a; Rowe *et al*., 2016). Applying the same simple regression to other published data reveals a similar significant relationship for 21 woody crop plantations in Germany (Fig. 2b; Walter *et al*., 2015). However, for land converted to *Miscanthus* cultivation in the UK, the relationship was not significant (Fig. 2b; *R* ^2^ = 0.06; *P* = 0.15), which might be attributable to the young age of the *Miscanthus* crops sampled (~7 years; Rowe *et al*., 2016). Soil carbon data from 135 Brazilian sugarcane crops planted on natural vegetation, grassland or cropland revealed a similar negative correlation, but only where soil clay content was below 60% (Fig. 2c; *R* ^2^ = 0.21, *P* = 0.02; Mello *et al*., 2014). For soils above 60% clay content, the range of ^pre^C was narrow relative to the other transitions, possibly confounding any relationships (Fig. 2d). In all studies, soil texture was a much weaker predictor of potential changes in soil carbon following LUC than ^pre^C (Mello *et al*., 2014; Walter *et al*., 2015; Rowe *et al*., 2016).

Despite evidence for a relationship between ∆C and ^pre^C, a limitation in most published studies is that soil carbon content following LUC is unlikely to have reached a new equilibrium as this may take several decades (Bárcena *et al*., 2014; Rowe *et al*., 2016). For locations where soil carbon losses have been observed, it is difficult to calculate the extent to which this carbon debt will be repaid over the life cycle of the crop based on current empirical data (Mello *et al*., 2014). However, from the available evidence, we conclude that targeting low carbon soils for perennial bioenergy crop cultivation will reduce soil carbon losses in the short‐term and promote soil carbon sequestration in the long‐term. Globally, it is proposed that managing land to promote such sequestration, and avoid loss, may be a valuable tool in the mitigation of climate change (Lal, 2003).

## Statement 3: Variability in soil carbon stock change influences the life‐cycle GHG balance of bioenergy production much more than variability in nitrogen‐related emissions over most common assessment timescales

At field‐scale, the impacts of annual cropland and grassland conversion to perennial bioenergy crops on soil carbon stocks and N_2_O emissions have been quantified under a variety of scenarios (location/crop/management), and the variability of responses across those scenarios has been described (Sections 1 and 2). However, it is important to interpret these field‐scale impacts in the context of the whole life cycle of energy production from biomass. Life cycle assessment (LCA) is a well‐established tool used to calculate the environmental impact of a product across a range of impact categories, including climate impacts, as compared to that of the conventional fossil‐based energy which would be displaced. Here, we used an LCA approach to estimate the GHG intensity (g CO_2_‐eq MJ^−1^) of four contrasting biofuel production scenarios (*Miscanthus*‐ethanol and SRC poplar‐renewable gasoline) based on reported ranges of ∆C (Qin *et al*., 2016), soil N_2_O emissions (based on our analysis shown in Fig. 1), and other life‐cycle emissions collected from the literature (see Appendix S1 for method).

**Figure 3:**
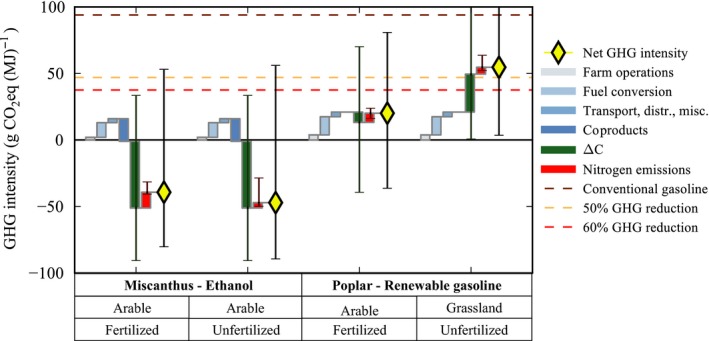
A life‐cycle perspective of the relative contributions and variability of soil carbon stock change and nitrogen‐related emissions to the net GHG intensity (g CO_2_‐eq MJ ^−1^) of biofuel production via select production pathways (feedstock/prior land‐use/fertilizer/conversion type). Positive and negative contributions to life‐cycle GHG emissions are plotted sequentially and summed as the net GHG intensity for each biofuel scenario, relative to the GHG intensity of conventional gasoline (brown line) and the 50% and 60% GHG savings thresholds (US Renewable Fuel Standard and Council Directive 2015/1513); orange and red lines, respectively. Default life‐cycle GHG source estimates are taken from Wang *et al*. (2012) and Dunn *et al*. (2013); direct N_2_O emissions from Fig. 1; and soil carbon stock change (0–100 cm depth) from Qin *et al*. (2016). See Appendix S1 for detailed methods.

Comparing fertilized and unfertilized crops grown on annual cropland and grassland, we found that the net GHG intensity of the biofuel scenarios varied widely from −39 to +54 g CO_2_‐eq MJ^−1^, but all delivered significant GHG savings compared to conventional gasoline (Fig. 3). Only bioenergy crops grown on annual cropland had a lower GHG intensity than the minimum 50% and 60% reduction thresholds (Fig. 3) mandated for ‘advanced’ and ‘cellulosic’ biofuels in the US Renewable Fuel Standard (110th Congress of the United States 2007) and for EU biofuel plants built after 2015 (Council Directive (EU) 2015/1513). Variability in net GHG intensity among the four biofuel scenarios was predominantly driven by significant differences in ∆C between *Miscanthus* and SRC poplar and in particular larger soil carbon losses for SRC poplar planted onto grassland (Qin *et al*., 2016). These values are consistent with a UK‐wide study of ∆C following LUC to bioenergy which reported significant gains on annual cropland and significant losses from grassland converted to perennial bioenergy crops (Richards *et al*., 2017).

The combined contribution of direct N_2_O and other nitrogen fertilizer‐related GHG emissions ranged from 4 to 11 g CO_2_‐eq MJ^−1^ (~4–12% of conventional gasoline reference emissions) (Fig. 3). These nitrogen emission estimates have a relatively wide range – with maximum estimates ~2–3x the median value, and minimum estimates close to zero – presumably driven in part by site‐level heterogeneity in climate and soil properties as explored in section 1. This suggests an opportunity for optimization through coordinated selection of planting locations and nitrogen application rates (Adler *et al*., 2012). Interestingly, while conversion of grasslands to bioenergy crops is characterized by high initial direct N_2_O emissions (Fig. 1), these transient responses are outweighed by the low postestablishment N_2_O emissions in unfertilized systems over the rest of the perennial crop planting cycle, and thus, total nitrogen impacts are dominated by differences in postestablishment emissions between fertilized and unfertilized systems. Overall the nitrogen‐derived contribution to the total fuel GHG intensity was similar or smaller in magnitude than emissions associated with biomass conversion to biofuel or coproduct crediting.

Comparing the best‐ and worst‐case scenarios for nitrogen emissions and ∆C for each biofuel scenario illustrates that differences in ∆C have a far greater influence on the GHG intensity of biofuels than nitrogen emissions (Fig. 3) when evaluated for a full 20‐year stand replacement cycle and calculated on a GWP_100_ basis. While soil carbon response will eventually attenuate, previous analysis suggests that this will often take many decades (Field *et al*., 2016), and it will take even longer for cumulative N_2_O emissions impacts to overtake cumulative soil carbon. Whilst these values represent the extremes, they demonstrate that site selection for bioenergy crop cultivation can make the difference between large GHG savings or losses, shifting life‐cycle GHG emissions above or below mandated thresholds. Reducing uncertainties in ∆C following LUC is therefore more important than refining N_2_O emission estimates (Berhongaray *et al*., 2017). Knowledge on initial soil carbon stocks could improve GHG savings achieved through targeted deployment of perennial bioenergy crops on low carbon soils (see section 2). In the UK, the bioenergy LUC model ELUM (Pogson *et al*., 2016; Richards *et al*., 2017) is an exemplar which could be replicated in other countries to predict the impacts of LUC on ∆C through to 2050 supporting this targeted deployment.

## Statement 4: Perennial bioenergy crops can provide substantial climate mitigation when used to replace fossil fuels but land‐use tensions must be mitigated

Significant reductions in GHG emissions have been demonstrated in many LCA studies across a range of bioenergy technologies and scales (Thornley *et al*., 2009, 2015). The most significant reductions have been noted for heat and power cases. However, some other studies (particularly on transport fuels) have indicated the opposite, that is that bioenergy systems can increase GHG emissions (Smith & Searchinger, 2012) or fail to achieve increasingly stringent GHG savings thresholds. A number of factors drive this variability in calculated savings, but we know that where significant reductions are not achieved or wide variability is reported there is often associated data uncertainty or variations in the LCA methodology applied (Rowe *et al*., 2011). For example, data uncertainty in soil carbon stock change following LUC has been shown to significantly influence the GHG intensity of biofuel production pathways (Fig. 3), whilst the shorter term radiative forcing impact of black carbon particles from the combustion of biomass and biofuels also represents significant data uncertainty (Bond *et al*., 2013).

Variations in LCA methodology or scope are equivalent to asking a different ‘LCA question’ (Adams *et al*., 2013) and can result in different GHG performance estimates for a given bioenergy system (Davis *et al*., 2009; Thornley *et al*., 2015). One significant source of methodological variation is in the assumptions around business‐as‐usual counterfactual scenarios for land use in LCA (Thomas *et al*., 2009; Achten *et al*., 2015). For example, significant GHG savings were achieved from bioenergy heat pathways utilizing agricultural residues and perennial bioenergy crops, but savings were extremely sensitive to the counterfactual land‐use scenario (Welfle *et al*., 2017). Essentially if establishment of the crop involved negative impingement on land used for food production there was a risk of a negative impact on the GHG balance. So, while there may be a desire to standardize methodologies to ensure ‘fair’ cross‐comparison, from a policy perspective it is important to ensure that the chosen methodology addresses the most relevant research question (Whittaker, 2014).

Identification of potential conflicts can help support the implementation of mitigation strategies such as using marginal or degraded land, and higher yielding, low‐input crops where appropriate. While there are some estimates of the availability of abandoned, degraded and marginal land (Campbell *et al*., 2008; Gu & Wylie, 2017), the production potential of dedicated bioenergy crops on such lands (Shield *et al*., 2012; Gelfand *et al*., 2013), and the relative value of land‐sparing vs. land‐sharing strategies (Anderson‐Teixeira *et al*., 2012) our understanding of system‐level performance trade‐offs is still limited (see section 6). Despite this knowledge gap, evidence does indicate that the use of low‐input perennial crops, such as SRC, *Miscanthus* and switchgrass, can provide significant GHG savings compared to fossil fuel alternatives provided that reasonable yields are obtained, low carbon soils are targeted (see sections 2 and 3 above), and the development context is one where tension with land use for food (and associated potential for iLUC emissions) is mitigated. There are many cases where these criteria are satisfied. It is, however, important that robust analysis of potential land‐use tensions is carried out using sensible yield assumptions. Legislative/policy focus may be on supply chains, and this has, to some extent, driven the concept of iLUC. However, in assessing the sustainability of bioenergy, it makes much more sense to view production of food and energy holistically and evaluate trade‐offs in land use at a much larger (global) scale (Njakou Djomo & Ceulemans, 2012). Increasing our knowledge of drivers of land‐use change and shifts in land management practice would therefore help us understand the likelihood of substantial climate mitigation being achieved.

## Statement 5: Perennial bioenergy crops marginally reduce water availability at landscape scale, but improve water quality through reduced nitrate leaching

Historical large‐scale shifts in land use from perennial grasslands and forests to annual croplands have resulted in less evapotranspiration and greater runoff and streamflow at the basin scale (Twine *et al*., 2004; Zhang & Schilling, 2006). Transition from annual crops to perennial grasses for energy production may again lead to significant perturbations to the hydrological cycle. Thus, the benefits of mitigating carbon emissions through perennial bioenergy feedstocks need to be evaluated against impacts they may cause on the hydrological cycle (Rowe *et al*., 2009) and on water quality as it relates to the nitrogen cycle (Castellano *et al*., 2010, 2013). *Miscanthus* and switchgrass, identified as promising feedstocks within the Midwestern US, fix more carbon from the atmosphere (Davis *et al*., 2010; Zeri *et al*., 2011, 2013; Anderson‐Teixeira *et al*., 2013) yet use the same (Hamilton *et al*., 2015) or marginally more water (Hickman *et al*., 2010; McIsaac *et al*., 2010; VanLoocke *et al*., 2010, 2012) than current annual crop agriculture. *Miscanthus* does, however, have the capacity to draw on deep soil water during a drought, potentially slowing the rate of recharge (Joo *et al*., 2017). Despite the increase in evapotranspiration associated with transitioning from annual to perennial crops, the increase in water use is almost universally accompanied by relatively greater increases in plant carbon uptake, leading to increased water use efficiency (VanLoocke *et al*., 2012; Zeri *et al*., 2013).

Significant reductions in leaching of dissolved inorganic nitrogen on a land surface basis are predicted to occur if land already growing maize for ethanol production is converted to a perennial feedstock (Davis *et al*., 2012; Iqbal *et al*., 2015). This reduction in leaching is attributed to lower fertilizer requirements, the continuous presence of a plant root sink for nitrogen, and the efficient internal recycling of nutrients by perennial grass species (Amougou *et al*., 2012; Smith *et al*., 2013). In support of this, *Miscanthus* and switchgrass assessed at a plot scale had significantly lower dissolved inorganic nitrogen leaching from subterranean drainage tiles relative to the typical maize/soy rotation, with fertilized plots of switchgrass showing little or no leaching after reaching maturity (Smith *et al*., 2013). Similarly, results from soil‐based measurements in the same feedstocks showed lower dissolved inorganic nitrogen relative to annual crops (McIsaac *et al*., 2010; Behnke *et al*., 2012). A recent meta‐analysis of the available literature concluded that switchgrass and *Miscanthus* had nine times less subsurface loss of nitrate compared to maize or maize grown in rotation with soya bean (Sharma & Chaubey, 2017). At the basin scale, displacement of maize production for ethanol by cellulosic perennial feedstock production could reduce total leaching by up to 22%, depending on the type of feedstock and management practice employed (Davis *et al*., 2012; Smith *et al*., 2013).

While these previous studies provide evidence for the potential ecosystem services of transitioning to cellulosic production, it is yet to be established what the total change to dissolved inorganic nitrogen export and streamflow would be under such scenarios. Hydrological processes are tightly coupled to the nitrogen cycle (Castellano *et al*., 2010, 2013), are key drivers of dissolved inorganic nitrogen transport through streams and rivers (Donner *et al*., 2002), and are sensitive to LUC (Twine *et al*., 2004). Various modelling scenarios, where current land cover over the Mississippi River Basin of the United States was altered to accommodate varying proportions of switchgrass or *Miscanthus,* showed that the impact on streamflow was small relative to the improvement in water quality (VanLoocke *et al*., 2017). While these results are for the Midwestern US, a region well suited for bioenergy production, the results indicate the importance of implementing a model framework explicitly validated to simulate the hydrology of various feedstocks at other locations suitable for bioenergy feedstock production.

## Statement 6: Ecosystem process‐based models are essential for assessing bioenergy viability and environmental performance at landscape and regional scales, but they have only recently been applied to evaluate specific land‐use policies and bioenergy deployment strategies

Much of the variability in the ∆C and N_2_O emissions observations explored in sections 1–3 is attributable to nonlinear responses of soil GHG fluxes to climate (Weier *et al*., 1993), soil texture (Bouwman *et al*., 2002) and land management intensity (Hoben *et al*., 2011). The use of models that represent ecosystem carbon, nitrogen and water dynamics via representations of physiochemical processes is essential for synthesizing the results of site‐specific, intensive and sometime contradictory field observations. Such models can then be used to extrapolate understanding of bioenergy crop performance across landscape and regional scales – with their associated spatial heterogeneity in soils, climate and land‐use patterns – to assess the production potential and environmental impacts of real‐world bioenergy systems.

Ecosystem process‐based models have been applied for spatially explicit bioenergy sustainability assessment for more than a decade (Sheehan *et al*., 2003). Modern high‐power computing enables ecosystem model application at the requisite fine spatial scales (Nichols *et al*., 2011) either through thousands of independent runs of ‘point’ models, for example DayCent (Davis *et al*., 2012; Yu *et al*., 2014; Field *et al*., 2016) and EPIC (Zhang *et al*., 2010a; Gelfand *et al*., 2013) or using ‘network’ models (e.g. SWAT; Wu *et al*., 2012; Gramig *et al*., 2013) that consider lateral hydrological or biogeochemical flows between networks of thousands of nodes. A review of spatially explicit, ecosystem process‐based model assessments of bioenergy systems published since 2010 (Table 1) shows that this technique has been applied to assess biomass yields and associated environmental impacts for a variety of bioenergy crops produced in various geographic areas, at scales covering almost three orders of magnitude. The studies with more modest geographic extent often quantified a larger number of environmental impacts or featured greater sophistication in terms of the richness of scenarios assessed and the degree of integration with LCAs and economic analyses (Table 1).

**Table 1:** Review of recent bioenergy landscape assessment studies detailing the bioenergy scenarios addressed, the environmental scope of the assessment and the degree of complexity and integration with external analyses

Study	Bryan *et al*. (2010)	Zhang *et al*. (2010b)	Wu *et al*. (2012)	Davis *et al*. (2012)	Gelfand *et al*. (2013)	Yu *et al*. (2014)	Gramig *et al*. (2013)	J.L. Field, S.G. Evans, E. Marx, unpublished data
*General scenario*								
Region	Lower Murray, Australia	SW Michigan, USA	James River Basin, USA	USA corn‐ areas	Midwest USA	Tennessee, USA	Wildcat Creek, Indiana, USA	SW Kansas, USA
Bioenergy crop	Wheat, canola	Various 1G and 2G crops	Corn, switchgrass	Switchgrass, *Miscanthus*	Variety incl. native successional	Switchgrass	Corn stover	Switchgrass
Model(s) used	APSIM (point)	EPIC (point)	SWAT (network) + EPIC (point)	DayCent (point)	EPIC (point)	DayCent (point)	SWAT (network) + DayCent (point)	DayCent (point)
Study area size (Mha)	11.9	0.98	5.35	~30	~156	~22	0.21	1.55
Characteristic spatial resolution^a^	~40 000 ha	ND^b^	~1500 ha	ND	ND	ND	ND	~410 ha
*Environmental scope*								
Biomass yields estimated?	X	X	X	X	X	X	X	X
Full soil GHG balance (inc N_2_O)?		X		X	X	X	X	X
Water quality or quantity considered?		X	X	X			X	
Erosion considered?		X					X	
*Systems‐level complexity & integration*								
Marginal lands explicitly considered?		X			X			X
Variable crop management?		X	X	X	X		X	X
Supply chain life cycle considered?	X	X			X	X		X
Economics feasibility considered?	X					X	X	X
Optimization algorithm applied?		X	X			X	X	X
Indirect leakage effects considered?								

a Total analysis area divided by number of unique model strata.

b Not determined.

While such methods are frequently used to account for the effects of spatial heterogeneity and management variability, their application to evaluate specific land‐use policies and low‐impact bioenergy deployment strategies remains much more limited (Table 1). For example, while feedstock production on marginal lands is an increasingly prominent strategy for minimizing iLUC and other unintended consequences, only a subset of the spatial assessment studies in Table 1 explicitly explored land quality, considered variable crop management intensity or integrated economic analysis in a manner capable of evaluating the production potential and practical viability of such a strategy.

Spatially explicit modelling can ideally synthesize field observations of bioenergy crop performance, fine‐scale correlations between land quality and land‐use history and empirical understanding of land management decisions (Rizzo *et al*., 2014; Skevas *et al*., 2016) to evaluate trade‐offs in the viability and environmental performance of specific feedstock cultivation siting choices. These insights can be integrated into higher‐level estimates of agricultural land availability and iLUC effects (Cohn *et al*., 2014; Hudiburg *et al*., 2016), and into coarser, global‐scale integrated assessment models (IAMs) that perform cross‐sectoral cost optimization analyses. This integration thus enables an assessment of the potential for bioenergy and BECCS to contribute to low‐cost GHG mitigation alongside competing energy technologies and other measures (Kriegler *et al*., 2013; Smith *et al*., 2016).

## Consensus and recommendations for future research

In the rush to pursue climate change mitigation strategies, the ‘carbon neutrality’ of bioenergy was not rigorously assessed. As more studies began to include assessment of dLUC and iLUC impacts, the credibility of first‐generation bioenergy as an environmentally sustainable, renewable energy source was damaged. In recent years, a more nuanced understanding of the environmental benefits and risks of bioenergy has emerged, and it has become clear that perennial bioenergy crops have far greater potential to deliver significant GHG savings than the conventional crops currently being grown for biofuel production around the world (e.g. corn, palm oil and oilseed rape). Furthermore, the increasingly stringent GHG savings thresholds for biofuels and bioenergy being introduced in Europe (Council Corrigendum 2016/0382(COD)) and the US (110th Congress of the United States 2007) are providing increased impetus for this transition to perennial bioenergy crops.

The assumption that N_2_O emissions from perennial crops strongly depend on the prior land use was largely borne out by the literature reviewed here (section 1). However, temporal hot spots of N_2_O emissions were identified in the establishment year in some locations when perennial grasses and woody crops were planted onto grassland, indicating an opportunity to further improve the GHG performance of bioenergy systems via N_2_O‐minimizing land preparation methods. With respect to soil carbon, increased confidence in the magnitude and variability of dLUC effects of perennial bioenergy feedstocks has been achieved through the development and application of robust measurement and modelling approaches (section 2). The assumption that annual cropland provides greater potential for soil carbon sequestration than grassland appears to be over‐simplistic, but there is an opportunity to improve predictions of soil carbon sequestration potential using information on the initial soil carbon stock as a stronger predictor of ∆C than prior land use. Further research is therefore warranted to determine whether these relationships between ^pre^C and ∆C, identified in the UK, Germany and Brazil, are consistent in other countries (section 2), and to reconcile this with ecosystem process‐based model approaches (section 6).

Considered in a whole life‐cycle context, these approaches have delivered robust evidence that bioenergy produced from dedicated perennial feedstocks can deliver significant GHG savings compared to fossil fuel systems (sections 3 and 4), as well as additional environmental benefits such as improved water quality (section 5). However, soil type, climate, prior land use and land management can significantly influence the net GHG intensity of perennial bioenergy crops (sections 1, 2, 3 and 6), and there is therefore a risk that not all bioenergy production pathways will deliver the GHG savings targeted in some renewable fuel policies (sections 3 and 4). Comparing the relative importance of nitrogen‐related emissions and ∆C suggests that reducing uncertainty of dLUC effects on soil carbon stocks is a higher priority than refining estimates of N_2_O emissions, where the effects of variance and uncertainty are less significant (for *Miscanthus*, SRC and switchgrass). This requires expanded observations to better understand ∆C with soil depth for deep‐rooting perennial crops and to extend the geographic reach of predictive models such as the ELUM model (Pogson *et al*., 2016; Richards *et al*., 2017), as well as general improvement of predictive models of LUC and management effects on ∆C.

There has been considerable progress in applying ecosystem process models at landscape and regional scales to account for spatial heterogeneity, though such techniques have only recently been adapted to assess the effectiveness of real‐world bioenergy technology deployments under specific feedstock supply strategies or land‐use policies (section 6). Such assessments are only as strong as the underlying model parameterization and validation efforts (Kang *et al*., 2010; Field *et al*., 2016). Bioenergy crop field trials that test productivity, soil carbon changes and N_2_O emissions across fine‐scale gradients of land quality are essential for assessing feedstock production on marginal lands but are still relatively rare (Shield *et al*., 2012; Boyer *et al*., 2013; Wilson *et al*., 2014; o Di Nasso *et al*., 2015; Roncucci *et al*., 2015). Equally important is the need to accurately capture past and future land management behaviour, based on landowner surveys or economic modelling, to determine which management practices are most likely under a given policy scenario. Once a solid foundation of ecosystem and land‐use modelling capabilities are in place, optimization techniques can be applied to help identify the lowest‐cost opportunities to improve GHG mitigation in bioenergy systems and to quantify trade‐offs with non‐GHG environmental impacts such as water use and water quality (sections 4 and 5).

## Conclusions

Optimal use of land is one of the global challenges of our generation as we attempt to derive a wide range of services from the land (food, feed, fibre, fuel, etc.) whilst also protecting biodiversity and preventing further environmental degradation (United Nations 2015, UNCCD 2017). At the same time, mitigation of climate change requires a wide range of reduction measures to be deployed globally if we are to keep warming below 2 °C (Pacala & Socolow, 2004; Smith *et al*., 2016). Bioenergy sits at the nexus of these two challenges as a potential tool to mitigate climate change which requires significant global LUC. Agriculture is one of the most environmentally disruptive of all human activities (Foley *et al*., 2011), and the fundamental question for bioenergy sustainability is whether opportunities for feedstock production can be identified that simultaneously minimize on‐site impacts (dLUC and N_2_O) and avoid displacing existing productive land uses that would likely result in compensatory agricultural expansion elsewhere (iLUC). Our analysis suggests that the direct impacts of dedicated perennial bioenergy crops on soil carbon and N_2_O are increasingly well understood, and are often consistent with significant lifecycle GHG mitigation from bioenergy relative to conventional energy sources. It is important that future work further verifies these outcomes for feedstock production on marginal lands to avoid displacement of existing crops, and that field observations and modelling results be synthesized into larger scale IAMs and other large‐scale modelling efforts to put the costs and benefits of large‐scale bioenergy deployment in a broader global context.

The research synthesized here demonstrates there is a mature and increasingly comprehensive evidence base on the environmental benefits and risks of bioenergy cultivation which can support the development of a diverse and sustainable bioenergy industry. It is critical for the future momentum of the bioenergy industry that the key areas of scientific consensus and our ability to quantify uncertainties on bioenergy carbon savings are clearly communicated, if we are to meaningfully support and engage in developing policies for sustainable bioenergy deployment which can contribute to the global goal of mitigating climate change.

## Supporting information


**Table S1.** Summary of scenarios in publications on soil N_2_O emissions from perennial bioenergy crops underlying Fig. 1.
**Table S2.** Annual average (mean) N_2_O emissions [Mg N_2_O ha^−1^ yr^−1^, mean ± SE (*n*)] calculated for four feedstocks individually.
**Appendix S1.** Method for calculation of net greenhouse gas (GHG) intensity for four biofuel production scenarios (illustrated in Fig. 3).
