# Supplementary material for: Consensus, uncertainties and challenges for perennial bioenergy crops and land use
Source: Glob Change Biol Bioenergy. 2017 Nov 27;10(3):150–64. doi: 10.1111/gcbb.12488 (PMC5815384; doi:10.1111/gcbb.12488)
Supplement: Supplementary file 1 — Table S1. Summary of scenarios in publications on soil N2O emissions from perennial bioenergy crops underlying Fig. 1. Table S2. Annual average (mean) N2O emissions [Mg N2O ha−1 yr−1, mean ± SE (n)] calculated for four feedstocks individually. Appendix S1. Method for calculation of net greenhouse gas (GHG) intensity for four biofuel production scenarios (illustrated in Fig. 3). [file GCBB-10-150-s001.docx]

**SUPPLEMENTARY INFORMATION**

**Table S1.** Summary of scenarios in publications on soil N_2_O emissions from perennial bioenergy crops underlying Figure 1.

| Current land-use | Prior land-use | Fertilizer rate  (kg ha^-1^ yr^-1^) | Location | Time since crop establishment (yrs) | Measurement method | Measurement frequency | Reference |
| --- | --- | --- | --- | --- | --- | --- | --- |
| *Miscanthus* | Annual crop | 0 | UK | 4-9 | Static | Monthly | Robertson *et al.* (2017) |
| *Miscanthus* | Annual crop | 0 | UK | 4-6 | Static | Monthly | Drewer *et al.* (2012) |
| *Miscanthus* | Annual crop | 0 | France | 22-23 | Static | Not known | Dufossé *et al.* (2014) |
| *Miscanthus* | Annual crop | 0, 80 | Germany | 8-9 | Static | Weekly | Gauder *et al.* (2012) |
| *Miscanthus* | Annual crop | 0, 63, 125 | Ireland | 17-18 | Static | Weekly | Roth *et al.* (2015) |
| *Miscanthus* | Annual crop | 0, 60, 120 | USA | 2-6 | Static | Twice-weekly | Davis *et al.* (2015) |
| *Miscanthus* | Annual crop | 0 | USA | 2-4 | Static | Bi-weekly | Smith *et al.* (2013) |
| *Miscanthus* | Annual crop | 0, 56 | USA | 2-4 | Static | Bi-weekly | Oates *et al.* (2016) |
| *Miscanthus* | Grassland | 0 | UK | 1-2 | Static | Monthly | McCalmont (2017) |
| *Miscanthus* | Grassland | 0 | UK | 2, 14 | Static | Weekly, bi-weekly | Roth *et al.* (2013) |
| *Miscanthus* | Grassland | 0, 75 | Denmark | 7 | Large static 6.4 m^3^ | Twice per day | Jorgensen *et al.* (1997) |
| Switchgrass | Annual crop | 0, 40, 120 | Canada | 3-4 | Static | Weekly, bi-weekly | Wile *et al.* (2014) |
| Switchgrass | Annual crop | 0, 56 | USA | 4-5 | Static | Bi-weekly | Duran *et al.* (2016) |
| Switchgrass | Annual crop | 0, 67 | USA | 5 | Static | Weekly | Schmer *et al.* (2012) |
| Switchgrass | Annual crop | 0, 56 | USA | 2-4 | Static | bi-weekly | Oates *et al.* (2016) |
| Switchgrass | Annual crop | 56 | USA | 2-4 | Static | Bi-weekly | Smith *et al.* (2013) |
| Switchgrass | Annual crop | 56, 112 | USA | 2 | Static | Bi-weekly | Nikièma *et al.* (2011) |
| Switchgrass | Annual crop | 0, 28, 56, 84, 112 |  | 2-4 | Static | 2-3 times per week | Ruan *et al.* (2016) |
| SRC willow | Annual crop | 0, 80 | Germany | 8-9 | Static | Weekly | Gauder *et al.* (2012) |
| SRC willow | Annual crop | 0, 75, 150 | Germany | 6-14 | Static | 4 times per week | Hellebrand *et al.* (2010) |
| SRC willow | Annual crop | 0, 75, 150 | Germany | 10-12 | Static | 4 times per week | Kavdir *et al.* (2008) |
| SRC willow | Annual crop | 0, 50, 75 | Germany | 0-2 | Static | 4 times per week | Balasus *et al.* (2012) |
| SRC willow | Annual crop | 0 | UK | 9-10 | Static | Monthly | Drewer *et al.* (2012) |
| SRC willow | Annual crop | 0 | UK | 8-9 | Static | monthly | McNamara, NP, pc |
| SRC willow | Grassland | 0 | UK | 4-7 | Static | Not known | Harris *et al.* (2017) |
| SRC willow | Grassland | 0 | USA | 0-1 | Static | Monthly | Nikiema *et al.* (2012) |
| SRC willow | Grassland | 0 | USA | 0-2 | Static | Monthly or bi-weekly | Palmer *et al.* (2014) |
| SRC poplar | Annual crop | 0, 75, 150 | Germany | 6-14 | Static | 4 times per week | Hellebrand *et al.* (2010) |
| SRC poplar | Annual crop | 0, 75, 150 | Germany | 10-12 | Static | 4 times per week | Kavdir *et al.* (2008) |
| SRC poplar | Annual crop | 0, 46 | Germany | 4-6, 1-3 | Static | Weekly | Walter *et al.* (2015) |
| SRC poplar | Annual crop | 0, 50, 75 | Germany | 0-2 | Static | 4 times per week | Balasus *et al.* (2012) |
| SRC poplar | Annual crop | 0, 40-50 | Germany | 1-4 | Automatic chambers | Campaign basis | Díaz-Pinés *et al.* (2017) |
| SRC poplar | Annual crop | 40 kg (yr 5) | Italy | 4-6 | Static | Monthly or bi-weekly | Sabbatini *et al.* (2016) |
| SRC poplar | Annual crop | 0 | USA | 3-22 | Static | Bi-weekly | Gelfand *et al.* (2016) |
| SRC poplar | Annual crop | 157, 213 (yr 3) | USA | 2-4 | Static | Bi-weekly | Oates *et al.* (2016) |
| SRC poplar | Annual crop | 0 | Belgium | 1-4 | Eddy covariance | Continuous | Zenone *et al.* (2016)  Zona *et al.* (2013) |
| SRC poplar | Grassland | 0 | USA | 0-1 | Static | Monthly | Nikiema *et al.* (2012) |
| SRC poplar | Grassland | 0 | USA | 0-2 | Static | Monthly | Palmer *et al.* (2014) |

**Table S2. Annual average (mean) N_2_O emissions (Mg N_2_O ha^-1^ yr^-1^, mean ± SE (n)) calculated for four feedstocks individually**

| Prior land-use | Feedstock | Fertilizer | Year 1 | Year 2 | Year 3+ |
| --- | --- | --- | --- | --- | --- |
| Annual | Miscanthus | Unfertilized |  | 0.57 ± 0.11 (3) | 0.19 ± 0.06 (7) |
| crops |  | Fertilized |  | 0.74 ± 0.12 (3) | 0.66 ± 0.11 (7) |
|  | Switchgrass | Unfertilized |  | 0.34 ± 0.11 (4) | 0.13 ± 0.04 (4) |
|  |  | Fertilized |  | 0.37 ± 0.07 (7) | 0.50 ± 0.11 (11) |
|  | SRC willow | Unfertilized | 0.19 (1) | 0.19 (1) | 0.13 ± 0.06 (5) |
|  |  | Fertilized | 0.26 ± 0.02 (2) | 0.26 ± 0.02 (2) | 0.37 ± 0.09 (5) |
|  | SRC poplar | Unfertilized | 0.79 ± 0.40 (4) | 0.34 ± 0.16 (4) | 0.36 ± 0.08 (6) |
|  |  | Fertilized | 0.25 ± 0.04 (3) | 0.50 ± 0.12 (5) | 0.50 ± 0.12 (8) |
| Grassland | Miscanthus | Unfertilized | 6.54 (1) | 1.15 ± 0.86 (2) | 0.11 ± 0.07 (2) |
|  |  | Fertilized |  |  | 0.33 (1) |
|  | Switchgrass | Unfertilized |  |  |  |
|  |  | Fertilized |  |  |  |
|  | SRC willow | Unfertilized | 4.80 ± 2.46 (7) | 1.02 ± 0.53 (6) | 0.07 (1) |
|  |  | Fertilized |  |  |  |
|  | SRC poplar | Unfertilized | 4.70 ± 1.92 (7) | 0.95 ± 0.46 (6) |  |
|  |  | Fertilized |  |  |  |

**Appendix S1. Method for calculation of net greenhouse gas (GHG) intensity for four biofuel production scenarios (illustrated in Fig. 3)**

Figure 3 shows the relative contributions and variance of soil carbon stock change (∆C) and nitrogen (N)-related GHG emissions to the total lifecycle footprint of biofuel production via several select production pathways (bioenergy crop, prior land-use, fertilizer regime, and conversion technology).

Default life-cycle GHG source estimates based on the Argonne National Laboratory GREET model are taken from Wang *et al.* (2012) Fig. 5 for the biochemical conversion of *Miscanthus* to ethanol, and from Dunn *et al.* (2013) Fig. 8 for the thermochemical conversion of poplar to renewable gasoline. We replaced default values for upstream N fertilizer production emissions by multiplying an emissions factor from GREET (3.04 kg CO_2_-eq kg N^-1^), the US national average for agricultural N fertilizer as per GREET.net v1.3.0.12842, database version 12707) by the median N fertilizer rate across the relevant field trials (61.5 and 48 kg N ha^-1^ yr^-1^ for fertilized *Miscanthus* production and fertilized poplar production, respectively). Emissions are translated from an area basis to a finished fuel basis based on representative area crop yields (10 and 11.8 Mg ha^-1^ yr^-1^ for *Miscanthus* and poplar, respectively, neglecting any changes in yield with N fertilizer application rate), and data on logistical losses, fuel yields, and fuel properties from the sources listed above.

Direct emissions from land use change (dLUC) are calculated using median, 5^th^ and 95^th^ percentile values for soil carbon stock change (0-100cm depth) as reported in Qin *et al.* (2016). Since our analysis focuses on feedstock production on marginal lands, we assume that iLUC leakage effects due to the displacement of existing agricultural production are negligible.

Minimum, median, and maximum observations of direct nitrous oxide (N_2_O) emission rates for years 1, 2, and 3+ after establishment were taken from Table 1 and combined to estimate total direct N_2_O emissions across a 20-year stand replacement cycle (median estimates shown in red in 2, with error bars representing the total range). Year 2 emissions were used as a stand-in for missing year 1 observations for fertilized *Miscanthus* production on annual crops land; likewise, year 3+ emissions for unfertilized poplar production on annual crops land were used as a stand-in for missing year 3+ observations of unfertilized poplar production on grassland. Indirect N_2_O emissions were estimated by multiplying the median dN_2_O totals by the ratio of IPCC Tier 1 iN_2_O-N and dN_2_O-N emission factors for a given mass of N applied as synthetic fertilizer or made available through soil organic matter mineralization (EF_iN2O-N_ = 0.00325 from Eqs. 11.9 and 11.10, and EF_dN2O-N_ = 0.01 from Eq. 11.2, resulting in a ratio of 0.325) (Eggleston *et al.* 2006). The resulting net GHG intensity for each biofuel pathway is plotted with a yellow diamond, as compared to a value of 94 g CO_2_-eq MJ^-1^ for conventional gasoline (red line) and 60% GHG saving threshold for cellulosic ethanol as in US Renewable Fuel Standard and Council Directive (EU) 2015/1513 (orange line).

**References**

Balasus A, Bischoff W-A, Schwarz A, Scholz V, Kern J (2012) Nitrogen fluxes during the initial stage of willows and poplars in short-rotation coppices. *Journal of Plant Nutrition and Soil Science,* **175**, 729-738.

Davis MP, David MB, Voigt TB, Mitchell CA (2015) Effect of nitrogen addition on *Miscanthus x giganteus* yield, nitrogen losses, and soil organic matter across five sites. *GCB Bioenergy,* **7**, 1222-1231.

Díaz-Pinés E, Molina-Herrera S, Dannenmann M*, et al.* (2017) Nitrate leaching and soil nitrous oxide emissions diminish with time in a hybrid poplar short-rotation coppice in southern Germany. *GCB Bioenergy,* **9**, 613-626.

Drewer J, Finch JW, Lloyd CR, Baggs EM, Skiba U (2012) How do soil emissions of N_2_O, CH_4_ and CO_2_ from perennial bioenergy crops differ from arable annual crops? *GCB Bioenergy,* **4**, 408-419.

Dufossé K, Drewer J, Gabrielle B, Drouet JL (2014) Effects of a 20-year old Miscanthus × giganteus stand and its removal on soil characteristics and greenhouse gas emissions. *Biomass and Bioenergy,* **69**, 198-210.

Dunn JB, Johnson M, Wang ZH, Wang M (2013) *Supply Chain Sustainability Analysis of Three Biofuel Pathways. Argonne National Laboratory USA*.

Duran BEL, Duncan DS, Oates LG, Kucharik CJ, Jackson RD (2016) Nitrogen fertilization effects on productivity and nitrogen loss in three grass-based perennial bioenergy cropping systems. *Plos One,* **11**,

Eggleston HS, Buendia L, Miwa K, Ngara T, Tanabe K (Eds.) (2006) *2006 IPCC Guidelines for National Greenhouse Gas Inventories,* Institute for Global Environmental Strategies.

Gauder M, Butterbach-Bahl K, Graeff-Hoenninger S, Claupein W, Wiegel R (2012) Soil-derived trace gas fluxes from different energy crops - results from a field experiment in Southwest Germany. *GCB Bioenergy,* **4**, 289-301.

Gelfand I, Shcherbak I, Millar N, Kravchenko AN, Robertson GP (2016) Long-term nitrous oxide fluxes in annual and perennial agricultural and unmanaged ecosystems in the upper Midwest USA. *Global Change Biology,* **22**, 3594-3607.

Harris ZM, Alberti G, Viger M, Jenkins JR, Rowe R, McNamara NP, Taylor G (2017) Land-use change to bioenergy: grassland to short rotation coppice willow has an improved carbon balance. *GCB Bioenergy,* **9**, 469-484.

Hellebrand H, Strähle M, Scholz V, Kern J (2010) Soil carbon, soil nitrate, and soil emissions of nitrous oxide during cultivation of energy crops. *Nutrient Cycling in Agroecosystems,* **87**, 175-186.

Jorgensen RN, Jorgensen BJ, Nielsen NE, Maag M, Lind AM (1997) N_2_O emission from energy crop fields of Miscanthus ''Giganteus'' and winter rye. *Atmospheric Environment,* **31**, 2899-2904.

Kavdir Y, Hellebrand HJ, Kern J (2008) Seasonal variations of nitrous oxide emission in relation to nitrogen fertilization and energy crop types in sandy soil. *Soil & Tillage Research,* **98**, 175-186.

McCalmont JP (2017) p.c.

Nikiema P, Rothstein DE, Miller RO (2012) Initial greenhouse gas emissions and nitrogen leaching losses associated with converting pastureland to short-rotation woody bioenergy crops in northern Michigan, USA. *Biomass & Bioenergy,* **39**, 413-426.

Nikièma P, Rothstein DE, Min D-H, Kapp CJ (2011) Nitrogen fertilization of switchgrass increases biomass yield and improves net greenhouse gas balance in northern Michigan, U.S.A. *Biomass and Bioenergy,* **35**, 4356-4367.

Oates LG, Duncan DS, Gelfand I, Millar N, Robertson GP, Jackson RD (2016) Nitrous oxide emissions during establishment of eight alternative cellulosic bioenergy cropping systems in the North Central United States. *GCB Bioenergy,* **8**, 539-549.

Palmer MM, Forrester JA, Rothstein DE, Mladenoff DJ (2014) Conversion of open lands to short-rotation woody biomass crops: site variability affects nitrogen cycling and N_2_O fluxes in the US Northern Lake States. *GCB Bioenergy,* **6**, 450-464.

Qin Z, Dunn JB, Kwon H, Mueller S, Wander MM (2016) Soil carbon sequestration and land use change associated with biofuel production: empirical evidence. *GCB Bioenergy,* **8**, 66-80.

Robertson AD, Whitaker J, Morrison R, Davies CA, Smith P, McNamara NP (2017) A Miscanthus plantation can be carbon neutral without increasing soil carbon stocks. *GCB Bioenergy,* **9**, 645-661.

Roth B, Finnan JM, Jones MB, Burke JI, Williams ML (2015) Are the benefits of yield responses to nitrogen fertilizer application in the bioenergy crop *Miscanthus × giganteus* offset by increased soil emissions of nitrous oxide? *GCB Bioenergy,* **7**, 145-152.

Roth B, Jones M, Burke J, Williams M (2013) The effects of land-use change from grassland to *Miscanthus x giganteus* on soil N_2_O emissions. *Land,* **2**, 437.

Ruan LL, Bhardwaj AK, Hamilton SK, Robertson GP (2016) Nitrogen fertilization challenges the climate benefit of cellulosic biofuels. *Environmental Research Letters,* **11**,

Sabbatini S, Arriga N, Bertolini T*, et al.* (2016) Greenhouse gas balance of cropland conversion to bioenergy poplar short-rotation coppice. *Biogeosciences,* **13**, 95-113.

Schmer MR, Liebig MA, Hendrickson JR, Tanaka DL, Phillips RL (2012) Growing season greenhouse gas flux from switchgrass in the northern great plains. *Biomass & Bioenergy,* **45**, 315-319.

Smith CM, David MB, Mitchell CA, Masters MD, Anderson-Teixeira KJ, Bernacchi CJ, DeLucia EH (2013) Reduced nitrogen losses after conversion of row crop agriculture to perennial biofuel crops. *Journal of Environmental Quality,* **42**, 219-228.

Walter K, Don A, Flessa H (2015) Net N_2_O and C_H_4 soil fluxes of annual and perennial bioenergy crops in two central German regions. *Biomass & Bioenergy,* **81**, 556-567.

Wang M, Han J, Dunn JB, Cai H, Elgowainy A (2012) Well-to-wheels energy use and greenhouse gas emissions of ethanol from corn, sugarcane and cellulosic biomass for US use. *Environmental Research Letters,* **7**,

Wile A, Burton DL, Sharifi M, Lynch D, Main M, Papadopoulos YA (2014) Effect of nitrogen fertilizer application rate on yield, methane and nitrous oxide emissions from switchgrass (*Panicum virgatum L.*) and reed canarygrass (*Phalaris arundinacea L.*). *Canadian Journal of Soil Science,* **94**, 129-137.

Zenone T, Zona D, Gelfand I, Gielen B, Camino-Serrano M, Ceulemans R (2016) CO_2_ uptake is offset by CH_4_ and N_2_O emissions in a poplar short-rotation coppice. *GCB Bioenergy,* **8**, 524-538.

Zona D, Janssens IA, Gioli B, Jungkunst HF, Serrano MC, Ceulemans R (2013) N_2_O fluxes of a bio-energy poplar plantation during a two years rotation period. *GCB Bioenergy,* **5**, 536-547.
